# Reversible cross-linking of gelatin by a disulphide-containing bis-succinimide for tunable degradation and release

**DOI:** 10.1016/j.fochx.2023.100699

**Published:** 2023-05-03

**Authors:** Shengbin He, Jingtong Wang, Zhao Li, Yongqiang Cao, Xueping Ning, Jian Sun, Quanzhi Chen, Min Ling

**Affiliations:** Key Laboratory of Longevity and Aging-related Diseases of Chinese Ministry of Education, Guangxi Colleges and Universities Key Laboratory of Biological Molecular Medicine Research, School of Basic Medical Sciences, Guangxi Medical University, Nanning, Guangxi 530021, PR China

**Keywords:** Gelatin, Cross-linking, Disulphide, Tunable degradation, Food engineering

## Abstract

Generally, gelatin was irreversibly cross-linked by chemical reagents to improve its water-resistance. However, few chemical reagents meet both the requirements of high cross-inking efficiency and tunable degradation. Here a reversible cross-linker, disulphide-containing bis-succinimide, was synthesized and used to control the cross-linking and degradation of edible gelatin film. Mixture of the gelatin and cross-linker for 120 min generated gelatin films that could preserve their morphology in 37 ℃ warm water for above 40 days. The gelatin film changed its microstructure from net to tightness after the cross-linking, thus facilitating the embedding of the targeted molecule into the gelatin material. The degradation of the cross-linked gelatin film and the release of its inclusion could be controlled by biocompatible glutathione. This work provides a good method for preparing modified gelatin with promising water-resistance, good biocompatibility, and tunable degradation for food/biomedical engineering applications.

## Highlights

• A reversible cross-linker was used to control the cross-linking and degradation of gelatin films.
• The cross-linked gelatin films could preserve their morphology in 37 ℃ warm water for above 40 days.
• The gelatin film changed its microstructure from net to tightness after the cross-linking.
• The degradation of the cross-linked gelatin film could be controlled by glutathione.

## Introduction

Collagen is the most abundant protein in animals. It is also the primary building block of our body’s skin, muscles, bones, and other connective tissues, thus providing a good resource for biomimetic materials preparation (Tang et al., 2022, Tumerkan et al., 2019). The main issue with the use of collagen lies in its antigenicity and poor reconstruction ability. As an incomplete hydrolysis product of collagen, gelatin exhibits no antigenicity and good reconstruction ability, and therefore has the potential to be used in food packaging, drug delivery, and tissue engineering (Casanova et al., 2020, Liu et al., 2017, Park et al., 2021, Stevenson et al., 2020, Zhang et al., 2020). However, native gelatin materials have poor water-resistance, as they are rapidly dissolved in solutions of physiological temperature (37℃).

Generally, the gelatin is cross-linked by chemical cross-linkers to improve its water-resistance and mechanical strength, thus facilitating its practical applications. Among the cross-linkers, aldehydes such as formaldehyde and glutaraldehyde are the most widely used due to their inexpensive cost and the ability to effectively cross-link gelatin molecules (Kwak et al., 2017, Maroufi et al., 2022, Zhu et al., 2019). However, aldehydes could cause cytotoxicity and adverse reproductive effects, and have recently been classified as carcinogens(Sun et al., 2021, Ye et al., 2021). To address this question, many biocompatible cross-linkers have been or are being developed, such as tannic acid (Aguzin et al., 2022, Muhoza et al., 2019), genipin (Teimouri et al., 2019, Yuan et al., 2019), dialdehyde polysaccharide (Kim, Jeong, Lee, Kim, & Jung, 2020), alginate dialdehyde (Park, et al., 2021), chlorine dioxide (He, Yin, Zhen, & Xu, 2015), ribose (Stevenson, et al., 2020), pullulan dialdehyde (Wang, Liu, Zhang, Zhang, & Zhu, 2019), carrageenan (Chen, et al., 2022), and methacrylate anhydride (Li, Zhang, Kawazoe, & Chen, 2017). Yet there is a stable/degradable paradox remained to be solved——the low degree of cross-linking leads to instability of the gelatin materials in warm water, while the high degree of cross-linking generates nondegradable gelatin materials (Huang, et al., 2019). An ideal cross-linking method should enable the users to control the preservation and degradation of the gelatin materials. For instance, in food packaging and drug delivery, the gelatin materials should have enough stability to resist water in vitro for long term storage; once inside the body, the gelatin material should be degradable under the physiological environment (Hanani, Roos, & Kerry, 2014). As far as we know, the cross-linking of gelatin mediated by most chemical reagents used today was irreversible, which leads to the stable/degradable paradox.

Here, we synthesized a disulphide-containing bis-succinimide (NHS-SS-NHS) for the reversible cross-linking of gelatin. The conditions where cross-linking reaction led to strong water-resistance of the gelatin material were identified. The disulphide was designed to cleave in response to glutathione in physiological conditions, followed by release of gelatin and its inclusion. Good biocompatibility of the cross-linked gelatin material was demonstrated. Hence, the preservation and degradation of gelatin materials can be controlled by using NHS-SS-NHS for food/biomedical engineering applications.

## Experimental section

### 2.1 Materials and reagents

Gelatin (Type B, 250 bloom) was purchased from Aladdin Bio-Chem Technology Co., LTD (Shanghai, China). Phosgene, 2-hydroxyethyl disulphide, tetrahydrofuran, *N*-hydroxysuccinimide, and Triethylamine were purchased from Sigma-Aldrich (St. Louis, USA). Phosphate-buffered saline (PBS), glutathione (GSH), Protein mark, and other electrophoresis chemicals were purchased from Solarbio Science&Technology Co, Ltd (Beijing, China). Dulbecco’s modified Eagle’s medium (DMEM) and other cell culture materials were purchased from Gibco (Gaithersburg, MD, USA) and used without further treatment. All other solvents used were of analytical reagent grade.

### 2.2 Synthesis of disulphide-containing bis-succinimide

**Fig. 1:**
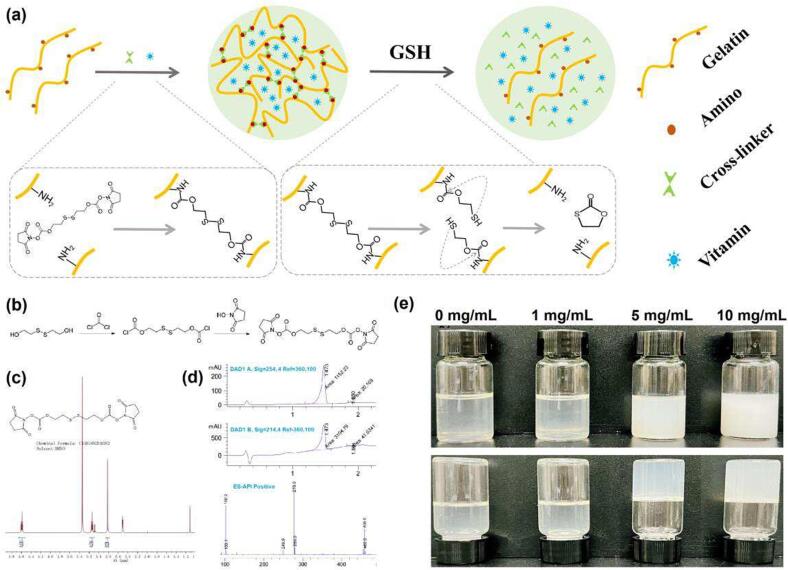
Reversible cross-linking of gelatin. (a) Schematic diagrams for Reversible cross-linking of gelatin by the disulphide-containing bis-succinimide cross-linker. (b) The synthesis of the cross-liner. (c) NMR analysis of the cross-linker. (d) LC-MS analysis of the cross-linker. (e) The cross-linker with final concentration ranging from 1 mg/mL to 10 mg/mL was mixed with gelatin solution (10%) at 37 °C.

The synthesis of disulphide-containing bis-succinimide is shown in Fig. 1a. Firstly, mix 10 mL phosgene (15 % in toluene) with 20 mL 2-hydroxyethyl disulphide (0.2 M in tetrahydrofuran) in a flask. The mixture was stirred for 10 h at room temperature. The solvents were removed through rotary evaporation. After that, 20 mL *N*-hydroxysuccinimide (0.08 g/mL in tetrahydrofuran) and 1000 μL triethylamine were added to the flask. The mixture was incubated at 40 °C to react for 18 h. The solvent was removed through rotary evaporation to obtain crude product, which was further purified by silica gel chromatography and recrystallized with icy hexane. Nuclear magnetic resonance spectroscopy (NMR) analysis and liquid chromatography tandem mass spectrometry (LC-MS/MS) analysis were carried out to confirm the chemical structure of the product.

### 2.3 Preparation of gelatin film

Glass substrates were used for preparing film mould, as described by our previous study (He, et al., 2015a). Specifically, the edges of a glass substrate were pasted with 10 layers of scotch tape, with thickness of 0.1 mm per layer, to form a groove of 30 mm (length) × 20 mm (width) × 1 mm (depth). Gelatin solutions were prepared by dissolving 0.1 g gelatin powder in 900 μL deionized water at 60 ℃. The cross-linker, NHS-SS-NHS (from 1 to 10 mg, dissolved in 100 μL DMSO), was added to the gelatin solution, and mix them well. The gelatin solution was cast into the glass groove by a steel knife to form a gelatin film with smooth surfaces and a thickness of 1 mm. The gelatin film was incubated 120 min at 25 ℃ for cross-linking reaction. After cross-linking, the gelatin film was soaked into 40 mL ethanol at 25 ℃ for 120 min. Finally, the gelatin films were dried for 60 min through natural evaporation, and kept in a desiccator for the subsequence use. The ethanol solution was subjected to LC-MS/MS analysis to quantify the unreacted NHS-SS-NHS. The amount of NHS-SS-NHS cross-linked with one gram of gelatin (Graft Rate, *GR*) was calculated via equation *GR*= (*A*_t_-*A*_u_)/*A*_g_, where *A*_t_, *A*_u_, and *A*_g_, represent the amount of total NHS-SS-NHS, the unreacted NHS-SS-NHS, and gelatin, respectively. When 1 mg/mL, 5 mg/mL, and 10 mg/mL NHS-SS-NHS were used for cross-linking, their *C*_r_ values were calculated to be 21 ± 3 μM/g, 84 ± 7 μM/g, and 132 ± 14 μM/g, respectively.

### 2.4 Mechanical strength testing

The Tensile strength, Elongation at break, and Young's modulus (GPa) of the gelatin films were determined by using a texture analyzer (TA. HD plus model, Stable MicroSystems, UK). The the thickness of the dry films was measured by the texture analyzer to be 85 ± 3 μm. The films were automatically cut by the analyzer into testing samples with an initial grip length of 15 mm and a width of 10 mm. The films were clamped and deformed by using a 100 N load cell with a cross-head speed of 10 mm/min until the films were broken.

### 2.5 Water-resistance assays

The water-resistance assay of the gelatin films was measured as follows. The gelatin films with different degrees of cross-linking were incubated in water at 37 ℃. The solution containing free gelatin molecules was sampled at various intervals for protein concentration determination, as described by Supplementary Protocol 1 and Supplementary Fig. S1. The film dissolution rate (*DR*) was calculated via equation *DR* = m_2_/m_1_ × 100 %, where m_1_ and m_2_ represent the initial mass of the gelatin film and the amount of free gelatin molecules in pure water, respectively. The contact angle measurements were carried out by using the sessile drop method on a goniometer (Dataphysics OCA50, Germany). First, a droplet of liquid (2 μL) was deposited on the film surface with a precision syringe. Then, the contact angle was measured at various contacting intervals, ranging from initial time point (≤2 s) to 45 s, on both sides of the drop. Six sample repeats were used to calculate their means and standard deviations. All the tests were conducted in chambers with a stable temperature of 25 ℃.

### 2.6 Scanning electron microscopy (SEM) and chemical composition analysis

SEM (FE-SEM SU8020, Japan) was used to observe the morphology of the cross-linked gelatin films at an accelerating voltage of 15 kV. The samples were coated with a thin layer of gold. The element composition of the gelatin films before and after cross-linking was determined by energy dispersion X-ray (EDX) analysis. FTIR (Thermo Scientific Nicolet iS10 FTIR, USA) was used to investigate chemical groups of the cross-linked gelatin. The spectra in the range of 400–4000 cm^−1^ with automatic signal gain were collected in 32 scans at a resolution of 4 cm^−1^ and divided by a background spectrum recorded from the clean empty cell at 25 ℃.

### 2.7 Molecular weight distribution

The molecular weight distribution of gelatin was investigated by both denaturing and non-denaturing sodium dodecyl sulfate–polyacrylamide gel electrophoresis (SDS-PAGE) according to our previous study (He, et al., 2015b). The sample was heated in boiling water for 5 min and analyzed by SDS-PAGE using 5% stacking gels and 6% resolving gels in a miniature electrophoresis unit (Bio-Rad Laboratories, Hercules, CA). The protein pattern was visualized by Coomassie Brilliant Blue R-250 staining. A protein marker with molecular weight ranging from 25 to 180 KDa was used to estimate the molecular weight of proteins.

### 2.8 Loading and release of small molecule from the cross-linked gelatin film

Vitamin B2, as a small molecule model, was loaded in the gelatin film by adding 6 mg vitamin B2 powder to 900 μL gelatin solution (11%). The mixture was ultrasounded (50kH) at 70 ℃ for 10 min to mix the vitamin B2 and gelatin well. Then, the cross-linker (from 1 to 10 mg, dissolved in 100 mL DMSO) was added to the above mixture. After incubation for 120 min, the mixture was cast into the glass groove by a steel knife to form a gelatin film as described in 2.3. To investigate the release of the vitamin B2 from the film, the prepared film (about 0.1 g) was soaked in 20 mL 37 ℃ water. Free vitamin B2 in the water was measured at frequent intervals by UV/VIS spectrophotometer at OD_480,_ as shown in Supplementary Fig. S2. For controls, GSH (final concentration of 10 mg/mL) was added to the solution to digest the film thoroughly, the release rate of which was taken as 100%.

### 2.9 Cells seeding and culture

Human renal epithelial cells (293 T) were cultured in DMEM medium supplemented with 10% fetal bovine serum (FBS, South America) in a humidified incubator (Thmorgan, Japan). Before cell seeding, gelatin films were sterilized under UV for 2 h, followed by immersion in sterile water for 60 min. Renew the water every 20 min, and then soak the films in 75% ethanol for 120 min. Finally, the gelatin films were washed with PBS for three times. The films were minced into circles of 10 mm in diameter and overlaid into the 6-well plates (Corning, USA). Then, the 293 T cells were seeded (5 × 10^4^ cells/cm^2^) on the films. To visualize the cell morphology, hematoxylin and eosin (HE) staining was performed after culturing the cells for 72 h.

### 2.10 Cell adhesion and cytotoxicity evaluation

Cell adhesion and cytotoxicity were evaluated by MTT assays as described in Supplementary Protocol 2. The cells were cultured 2 to 4 h for cell adhesion evaluation, and 1 to 3 days for cell cytotoxicity assay.

### 2.11 Cellular apoptosis analysis

The cellular apoptosis was characterized by using Annexin V-FITC/PI apoptosis kit combined with a flow cytometer (BD Bioscience, USA). The 293 T cells were seeded in the 6-well plates at a concentration of about 50,000 cells per well. After culturing for 72 h, the cells were digested by trypsin and washed off by PBS. The cells were collected by centrifugation at 1500 rpm for 5 min. Then, the cells were resuspended by 500 µL binding buffer, followed by adding 5 µL Annexin V-FITC and 5 µL propidine iodide (PI) to the cellular solution. The mixture was incubated for 5 min at room temperature, and then subjected to apoptosis analysis by using the flow cytometer combined a FACSDiva software.

## 3 Results and discussion

### 3.1 Principle of reversible cross-linking of gelatin by the disulphide-containing bis-succinimide

Fig. 1a shows the design of the cross-linker and how it reacts with the gelatin. The two terminals of the cross-linker were attached with succinimide-activated carboxyl groups, which react with the amino groups of the gelatin molecules to form cross-linked product. After cross-linking, small molecular compounds such as vitamins could be packaged in the gelatin for nutrient loading. The disulfide linkage was designed to cleave in response to glutathione (GSH), followed by the release of native gelatin molecules through a self-immolative reaction(Xu et al., 2012, Riber et al., 2015)；thus the cargoes in the gelatin were also released. Fig. 1b shows the diagram for the synthesis of the disulphide-containing bis-succinimide (NHS-SS-NHS) cross-linker. The chemical structure of cross-linker was verified by NMR and LC-MS, as shown in Fig. 1c and Fig. 1d, indicating the successful synthesis of the cross-linker. The mixture of 10% gelatin solution with 5 mg/mL cross-linker led to solidification of the solution within 120 min, as shown in Fig. 1e, indicating the rapid and effective cross-linking reaction between the gelatin and the cross-linker.

### 3.2 Water-resistance of the cross-linked gelatin films

**Fig. 2:**
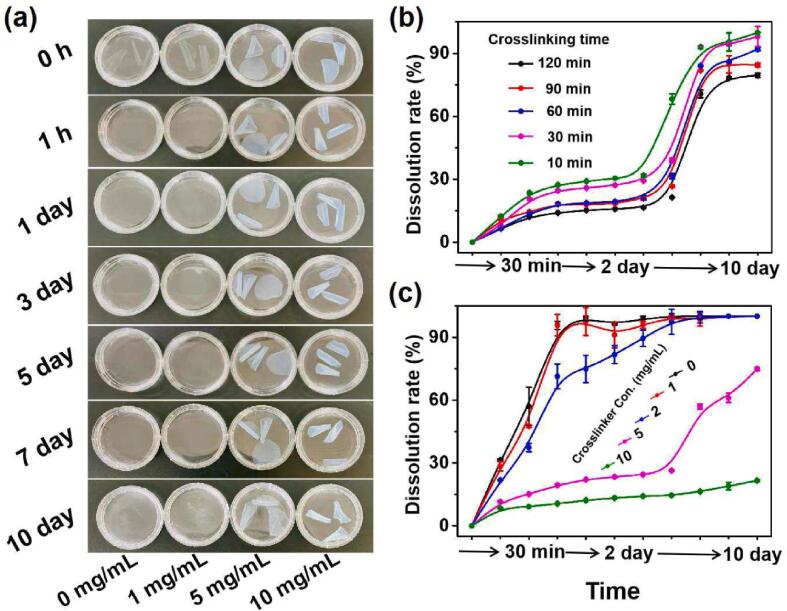
Water-resistance of the cross-linked gelatin films. (a) Shapes of the gelatin films in warm water, with cross-linker concentration ranging from 0 to 10 mg/mL. The films were cross-linked for 120 min and soaked in 37 °C water, and the pictures were taken at various soaking intervals. (b) Dissolution kinetics of the gelatin films in warm water, with a cross-linker concentration of 5 mg/mL, and cross-linking times ranging from 10 min to 120 min. (c) Dissolution kinetics of the gelatin films in warm water, with a cross-linking time of 120 min and cross-linker concentrations ranging from 0 to 10 mg/mL. The dissolution rate was defined as the percentage of free gelatin molecules dissolved into the water.

Gelatin films cross-linked by NHS-SS-NHS at different concentrations were soaked in 37 °C water to investigate their water-resistance. As shown in Fig. 2a, gelatin films without cross-linking were completely dissolved in water within 1 h. The cross-linking degree of the films increased with the increasing of NHS-SS-NHS concentration; as a result, the preservation time of the films in water increased. When the NHS-SS-NHS concentration increased to 10 mg/mL, the films could keep their morphology undamaged in warm water for above 40 days (Supplementary Fig. S3). Fig. 2b and Fig. 2c show the dynamic dissolution of the gelatin films, of which the cross-linking degree was controlled by changing the cross-linking time (Fig. 2b) and cross-linker concentration (Fig. 2c). As the cross-linking reaction was fast, it was more favorable to adjust the cross-linker concentration than cross-linking time for controlling the degree of cross-linking. For gelatin films cross-linked by 10 mg/mL NHS-SS-NHS, only 17% of gelatin molecules were dissolved in warm water after 15 days incubation. The results indicated the degree of cross-linking and the water-resistance of the gelatin films could be controlled to the extent where it was needed.

### 3.3 Mechanical properties and hydrophobicity of the gelatin films

**Fig. 3:**
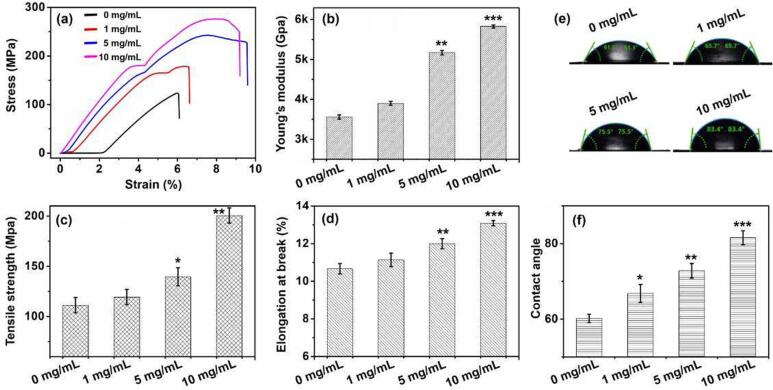
Mechanical properties and hydrophobicity of the gelatin film. (a) Representative stress–strain curves of the gelatin films. (b-d) Comparisons in Young’s modulus, Tensile strength, and Elongation at break between films of different cross-linking degrees. (e-f) Comparisons in water contact angle between films of different cross-linking degrees (the contact angle was detected at the initial time point (≤2 s). The differences between the control groups (0 mg/mL cross-linker) and the cross-linked groups were indicated by the asterisks, with one asterisk for p < 0.05, two asterisks for p < 0.01, and three asterisks for p < 0.001.

Fig. 3a shows the representative tensile stress–strain curves of the gelatin films before and after cross-linking. Based on the curves, the Young's modulus, Tensile strength, and Strain at break were calculated as shown in Fig. 3b-3d. The stretchability of the films was proportional to the NHS-SS-NHS concentration used for cross-linking. When 10 mg/mL NHS-SS-NHS was used, the Young’s modulus of the films was increased by 55 ± 1 %, Tensile strength by 82 ± 6 %, and Elongation at break by 24 ± 1 %. These results indicated the cross-linked gelatin films had much better mechanical properties than that of non cross-linking ones.

Compared with native gelatin, the NHS-SS-NHS was hydrophobic. Theoretically, the presence of NHS-SS-NHS would increase the hydrophobicity of the gelatin film. If a film is more hydrophilic, the water is more likely to wet the film surface and its contact angle will be smaller (Kchaou, Jridi, Benbettaieb, Debeaufort, & Nasri, 2020). Therefore, we use water contact angle to evaluate the surface wettability of the films and to further evaluate the cross-linking degree of gelatin films, as shown in Fig. 3e-f and Supplementary Fig. S4. With the increase of cross-linker from 1 mg/mL to 10 mg/mL, the contact angle at the initial time point increased from 65.7° to 83.4°, indicating more hydrophobic groups were present on the surface of the gelatin film. The improvements in mechanical strength and hydrophobicity of the gelatin films favor the food storage and transportation in packaging applications (A. Zhang, Han, & Zhou, 2023).

### 3.4 FTIR analysis and molecular-weight distribution

**Fig. 4:**
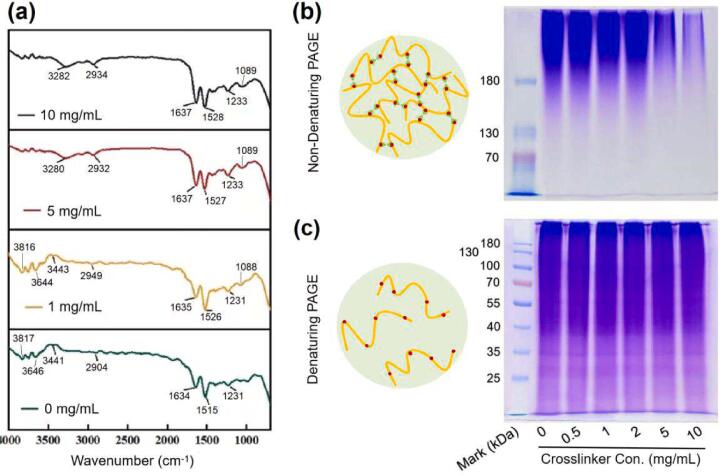
FTIR analysis and molecular-weight distribution of the gelatin film. (a) FTIR spectra for gelatin films of different cross-linking degrees. (b-c) non-denaturing (b) and denaturing (c) electrophoresis images of gelatin molecules extracted from the cross-linked gelatin films.

The FTIR spectra of gelatin films before and after cross-linking are shown in Fig. 4a. The absorption peaks of amide bonds for native gelatin were located at 1634, 1515, and 1231 cm^−1^, corresponding to amide-I, amide-II, and amide-III, respectively (Ahammed et al., 2021, Shi et al., 2020). The cross-linking by NHS-SS-NHS generated bathochromic shifts of these three peaks due to the presence of electron-attracting groups (-O-CO-NH-) of the cross-linker. The ester bond (–CH_2_-O-CO- (Wang, et al., 2019)) introduced into the gelatin by the cross-linker was prominent at 1089 cm^−1^, which was absence in native ones. The absorption at 3651–3824 cm^−1^ of native gelatin corresponds to the stretching vibrations of O—H in hydroxyl containing amino acids (Bilici, Tatar, Senturk, Dikyol, & Koc, 2022); after cross-linking, this absorption was weakened by the abundant carbon-hydrogen bonds (–CH_2_-, at 2934 cm^−1^ (Bilici, et al., 2022)) and nitrogen–hydrogen bonds (–NH-, at 3281 cm^−1^ (Pilipenko, et al., 2019)) of the cross-linker. These results proved that the gelatin molecules were successfully cross-linked by NHS-SS-NHS through covalent bonds.

Fig. 4b and 4c show the polyacrylamide gel electrophoresis (PAGE) of the gelatin molecules extracted from the cross-linked films. Because the molecular weights of the gelatin were heterogeneous, there were no separated protein bands in the polyacrylamide gel, as reported previously (He, et al., 2015b; Jus, et al., 2012). For non-denaturing PAGE, as the NHS-SS-NHS increased, more and more gelatin molecules were linked to formed solid matter, which could not enter the holes of polyacrylamide gel. As a result, the amount of gelatin molecules in the gel decreased gradually (Fig. 4b). When the cross-linked gelatin was denatured by mercaptoethanol, free gelatin molecules were released and there was no difference in electrophoresis results between gelatin films of different cross-linking degrees (Fig. 4c). These results indicated the cross-linking of gelatin by NHS-SS-NHS could be reversed by reductive reagents.

### 3.5 Microstructure and elemental composition analysis of the gelatin film

**Fig. 5:**
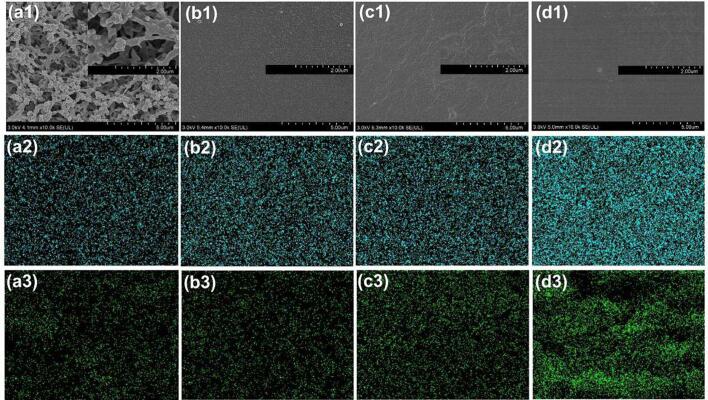
Microstructure and elemental composition analysis of the gelatin films. (a-d) Representative gelatin films cross-linked by 0 mg/mL, 1 mg/mL, 5 mg/mL, and 10 mg/mL cross-linker, respectively. (a1-d1) Microstructure of the gelatin films without water. (a2-d2) Oxygen distribution of the films. (a3-d3) Sulphur distribution of the films.

Fig. 5 shows the scanning electron microscope images and elemental composition of the gelatin films at various levels of cross-linking. The gelatin film without cross-linking exhibited a network structure (Fig. 5a1), as described by Pei et al. (Pei, et al., 2021). The network structure collapsed rapidly upon warm water due to the poor water-resistance of the native gelatin (Supplementary Fig. S5a). The addition of NHS-SS-NHS increased the density of the gelatin networks to the extent where all the micropores were enclosed (Fig. 5b1-d1), which was ascribed to the tight coupling of the gelatin molecules. This tight coupling protects the film from being destroyed by water (Supplementary Fig. S5b-d)). The changes in elemental composition between different films were analyzed by EDX. As shown in Fig. 5a2-d2 and Fig. 5a3-d3, both oxygen and sulphur densities increased with the increasing of the cross-linker. Because the percentage of both oxygen and sulphur in the cross-bridge was 30.8 wt%, which was higher than that of gelatin. Overall, these results were in qualitative agreement with the observations from tensile testing, water contact angle assays, and FTIR analysis.

### 3.6 Tunable loading and release of small molecule from the cross-linked gelatin film

**Fig. 6:**
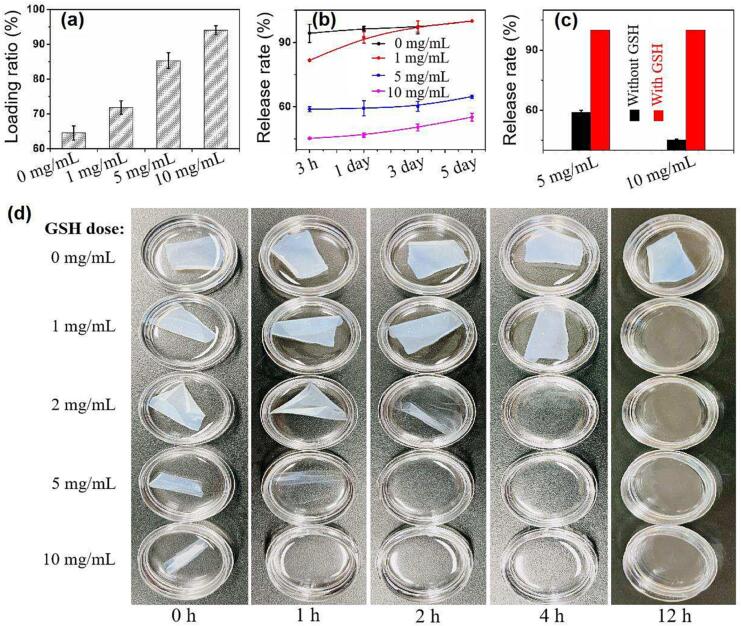
Tunable loading and release of vitamin B2 from the cross-linked gelatin film. (a) Vitamin B2 loading ratio for films cross-linked by NHS-SS-NHS of different concentrations. Loading ratio was defined as the percentage of vitamin B2 embedded into the film. (b) Release of vitamin B2 into 37 °C water from the films cross-linked by NHS-SS-NHS of different concentrations at various time points. (c) Release of vitamin B2 into the water from the films in the presence and absence of GSH. GSH of 2 mg/mL was used. (d) Degradation of the films in 37 °C water under different GSH concentrations. The films were cross-linked by 10 mg/mL NHS-SS-NHS.

Many small molecular nutrients, such as vitamin B2 and vitamin D are sensitive to photodegradation, and embedding them into gelatin film can favor the preservation and transportation of functional foods. Here we used Vitamin B2 as a model to investigate the loading and release behavior of small molecules from the gelatin films. As shown in Fig. 6a, the vitamin B2 loading ratio of the native gelatin film is relatively low (64%) due to the porous structure of the film. The enhanced rigidity of the cross-linked gelatin is very helpful in embedding vitamin B2 into the film, with a loading rate increased to 95%. When the films were soaked in warm water, the loaded vitamin B2 released parallel to the degradation of gelatin film, as shown in Fig. 6b and Fig. 2a. Hence, without cross-linking, all the vitamin B2 molecules were released within three hours. For films cross-linked by 10 mg/mL NHS-SS-NHS, more than half of the vitamin B2 was still entrapped in the film even after 5 days’ soaking. The complete release of vitamin B2 could be controlled by adding 2 mg/mL GSH, as shown in Fig. 6c. The gelatin films were subjected to degradation in PBS containing GSH of different concentrations to further investigate the effects of GSH dose on the degradation rate of gelatin films, as shown in Fig. 6d. There was a dose dependent effect of GSH on the degradation of gelatin film. When the GSH concentration reached to 2 mg/mL, the films were dissolved completely within 4 h. Therefore, the loading and release of small molecules in the gelatin material could be controlled by using NHS-SS-NHS combined with GSH.

### 3.7 Cell adhesion and cytotoxicity evaluation

The cross-linking by NHS-SS-NHS would consume large amounts of amino groups of the gelatin, which may inactivate its sites for cell adhesion and hamper its applications in tissue engineering. Hence, 293 T cells were incubated with gelatin films of different cross-linking degrees to evaluate the cell adhesion efficiency. When 0–1 mg/mL NHS-SS-NHS was used, the gelatin was dissolved rapidly at physiological temperature, and the cells adhered to polystyrene plates (TCPS) were taken as controls. As shown in Supplementary Fig. S6a, compared with TCPS, the cross-linked gelatin films exhibited advantages in promoting cell adhesion, indicating the cellular adhesion sites of the film were undistorted. Supplementary Fig. S6b shows the proliferation results of 293 T cells on the cross-linked gelatin films for cytotoxicity evaluation. The increases of the cells in a period of 3-day culture indicated that the cell expansion took place on the cross-linked gelatin film. There was a positive relationship between the proliferation rate and the NHS-SS-NHS dose used for cross-linking. We ascribe this phenomenon to the stable adhesion of cells to the highly cross-linked film. Previous studies had demonstrated an increased growth and adhesion of the cells grown on the gelatin matrix, which is a highly biocompatible material with surface anchor points (Yao, Zhang, Luan, & Lin, 2012; He, et al., 2015b). Lower NHS-SS-NHS doses produced lower degrees of cross-linking; as a result, the films were more likely to dissolve to lose cellular adhesion sites that were prerequisite for cell proliferation. Photos of cells were taken by an inverted microscope combined with eosin staining. As shown in Supplementary Fig. S6c, the cells grew on TCPS and gelatin films were bright with fusiform and circular, and no morphological differences were observed between the films of different cross-linking degrees.

### 3.8 Cellular cycle and apoptosis assays

Supplementary Fig. S7 shows the cellular cycle of the cells grown on the film. There was no significant difference in distribution of the cells in G1 phase, S phase, and G2 phase between the TCPS and the gelatin films, indicating the cross-linker did not interfere the DNA synthesis and cell division. AnnexinV-FITC/PI double staining was used to detect the apoptotic cells after proliferation for 72 h on the cross-linked gelatin films. As shown in Supplementary Fig. S8, the dead cells for 5 mg/mL and 10 mg/mL cross-linker were limited to 5%, which was significantly (p < 0.01) lower than that of control and 1 mg/mL cross-linker. Therefore, the addition of NHS-SS-NHS could not induce apoptosis to the cells; instead, it could increase the viability of cells by stabilizing the adhesion sites of gelatin film as observed in cell adhesion and cytotoxicity assays. These results demonstrated good biocompatibility of the cross-linked gelatin material, which could provide frameworks for cell adhesion and proliferation in tissue engineering and organ culture.

## 4 Conclusion

In this study, reversible cross-linking of gelatin was first put forward by using a laboratory-synthesized disulphide-containing bis-succinimide (NHS-SS-NHS) to reconstruct edible film. The degree of cross-linking could be controlled by changing the NHS-SS-NHS dose from 1 mg/mL to 10 mg/mL. Good performances of the cross-linked gelatin film with respect to water-resistance, mechanical strength, hydrophilicity, and structural tightness were demonstrated. The reversible cross-linking enables the users to embed small molecules into the gelatin film efficiently as well as to control the degradation of the film and release of the loaded targets by adding glutathione. Cell adhesion, cytotoxicity, cellular cycle, and apoptosis assays confirmed good biocompatibility of the cross-linked gelatin film. The reversible cross-linking of gelatin material by NHS-SS-NHS could be employed to control the degradation of the material and the release of its inclusion for food/biomedical engineering applications.

## CRediT authorship contribution statement

**Shengbin He:** Conceptualization, Investigation, Writing – review & editing. **Jingtong Wang:** Investigation, Writing – review & editing. **Zhao Li:** Methodology, Data curation. **Yongqiang Cao:** Methodology, Data curation. **Xueping Ning:** Methodology, Data curation. **Jian Sun:** Methodology, Data curation. **Quanzhi Chen:** Project administration, Funding acquisition, Writing – review & editing. **Min Ling:** Project administration, Funding acquisition, Writing – review & editing.

## Declaration of Competing Interest

The authors declare that they have no known competing financial interests or personal relationships that could have appeared to influence the work reported in this paper.

## Data Availability

Data will be made available on request.
