# Supplementary material for: Reversible cross-linking of gelatin by a disulphide-containing bis-succinimide for tunable degradation and release
Source: Food Chem X. 2023 May 3;18:100699. doi: 10.1016/j.fochx.2023.100699 (PMC10195852; doi:10.1016/j.fochx.2023.100699)
Supplement: Supplementary data 1 [file mmc1.docx]

**Supporting Material for**

**Reversible cross-linking of gelatin by a disulphide-containing bis-succinimide for tunable [degradation](javascript:;) and release**

Shengbin He^*, †, a^, Jingtong Wang^†, a^, Zhao Li^a^, Yongqiang Cao^a^, Xueping Ning^a^, Jian Sun^a^, Quanzhi Chen^*, a^, Min Ling^*, a^

^a^ Key Laboratory of Longevity and Aging-related Diseases of Chinese Ministry of Education, Guangxi Colleges and Universities Key Laboratory of Biological Molecular Medicine Research, School of Basic Medical Sciences, Guangxi Medical University, Nanning, Guangxi 530021, P. R. China.

^†^These authors contributed equally to this work.

*To whom correspondence should be addressed. E-mail: [comhsb@163.com](mailto:comhsb@163.com) (S. He); [quanzhi_chen@163.com](mailto:quanzhi_chen@163.com) (Q. Chen); [lingmin70@163.com](mailto:lingmin70@163.com) (M. Ling)

This Supporting Information inlcudes Supplementary Figure S1, Figure S2, Figure S3, Figure S4, Figure S5, Figure S6, Figure S7, Protocol S1, and Protocol S2.


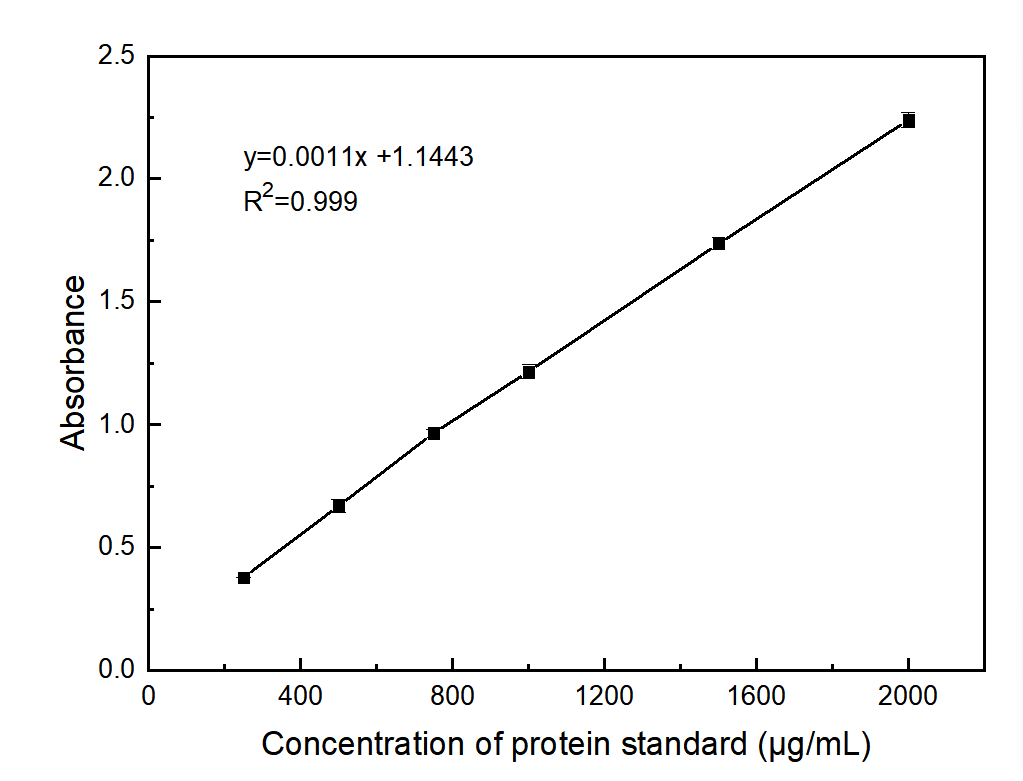


**Supplementary Fig. S1.** Dependence of the absorbance at 562 nm on the concentration of free gelatin molecule detected by bicinchoninic acid absorbance assay.


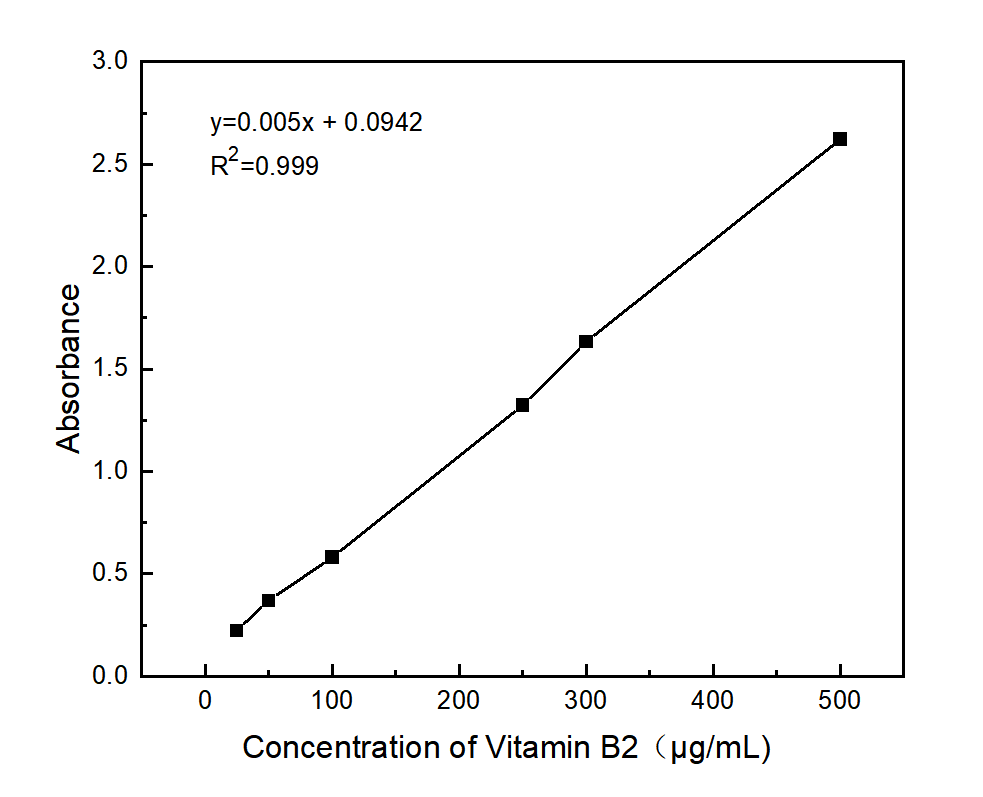


**Supplementary Fig. S2.** Dependence of the absorbance at 480 nm on the concentration of free Vitamin B2.


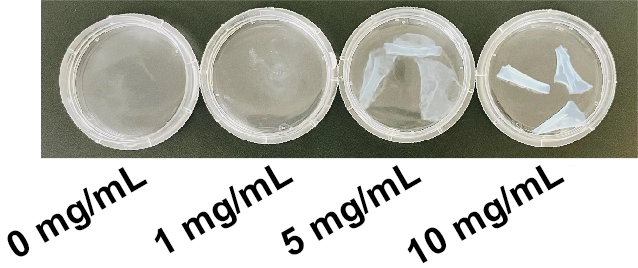


**Supplementary Fig. S3.** 1. Shapes of the gelatin films soaked in warm water for 40 days, with cross-linker concentration ranging from 0 to 10 mg/mL.


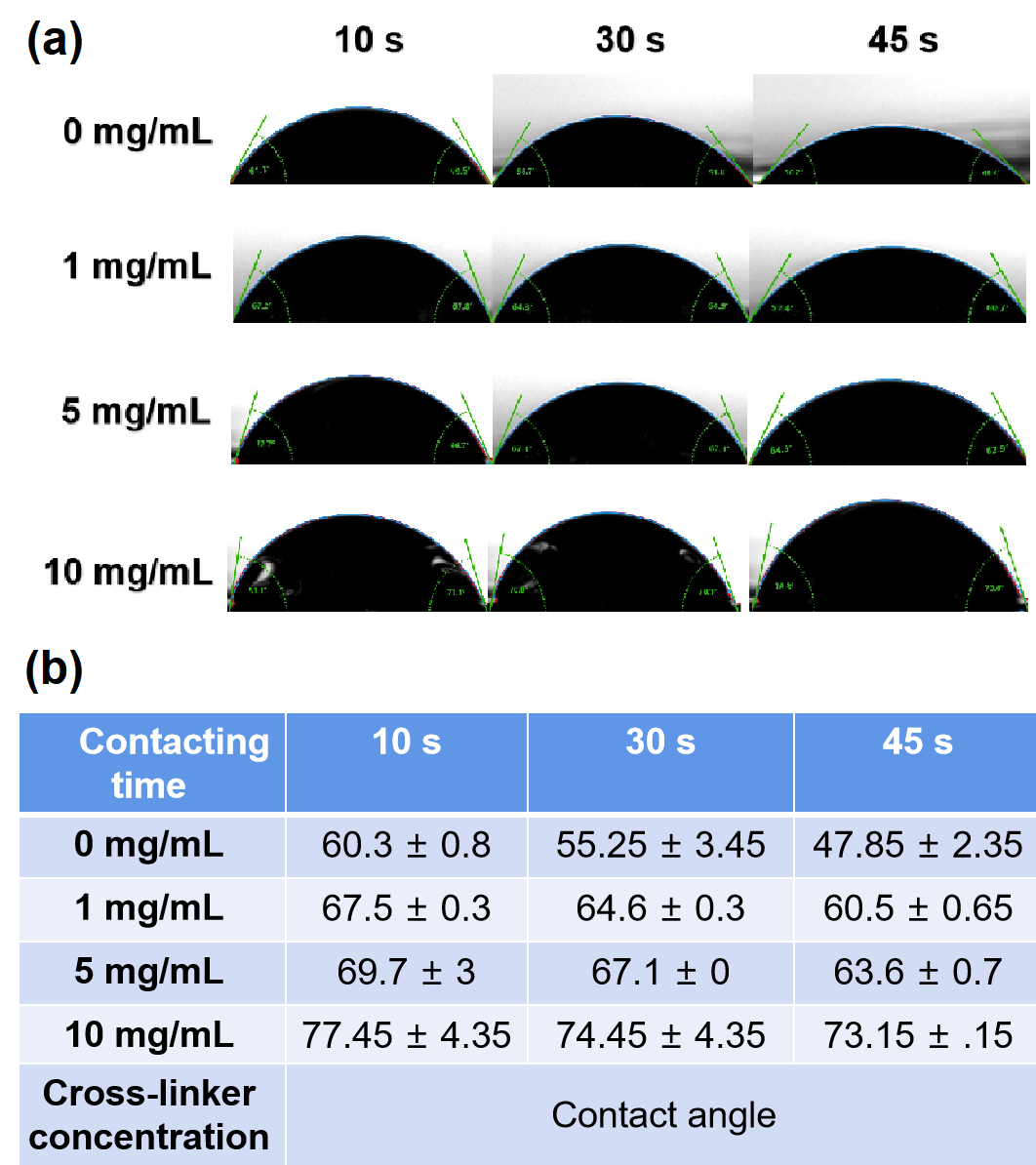


**Supplementary Fig. S4.** Comparisons in water contact angle between films of different cross-linking degrees. (a) Representative images captured at various contacting time (from 10 s to 45 s). (b) Average value for six repeats.


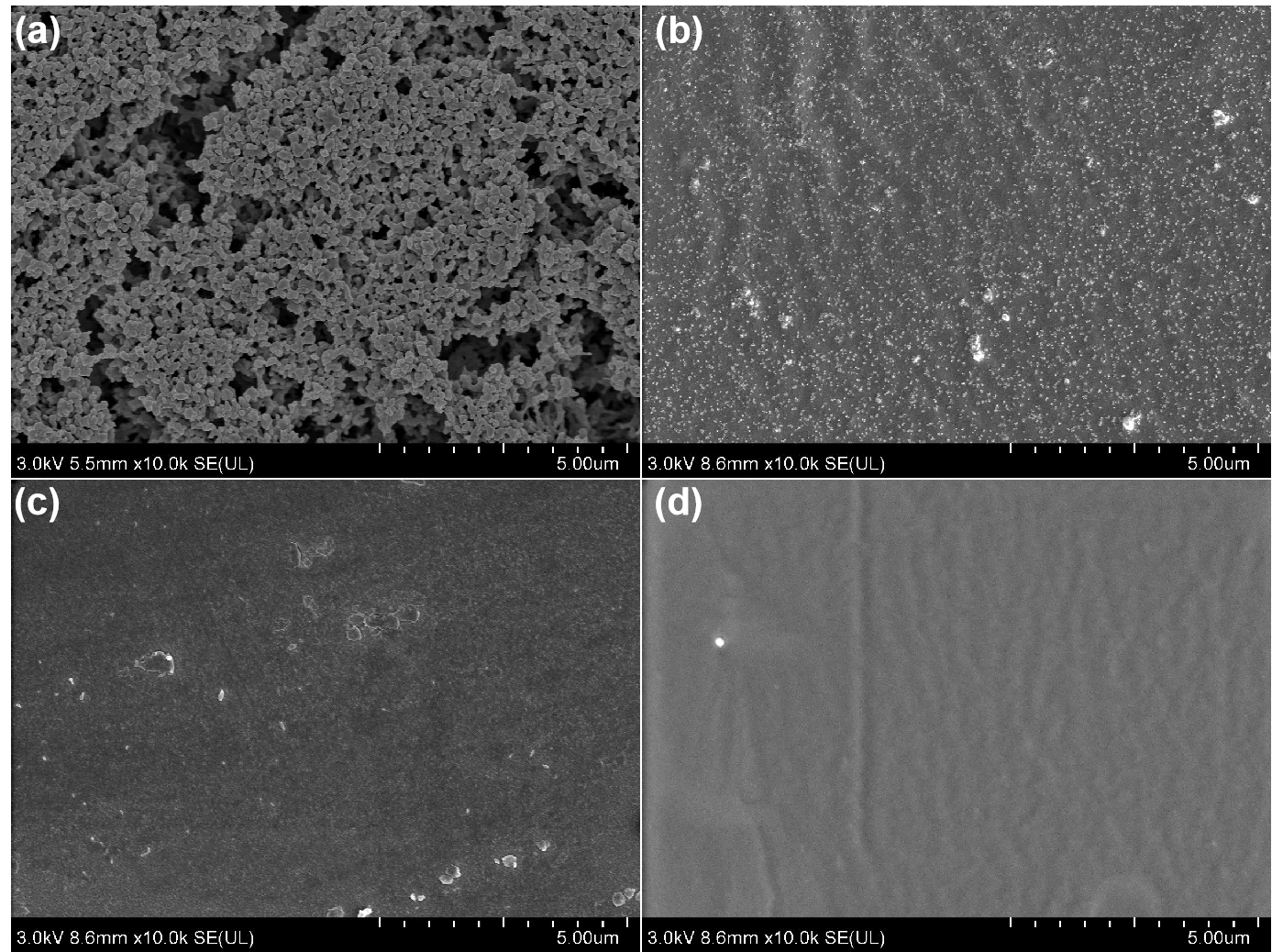


**Supplementary Fig. S5.** Microstructure of the gelatin films after contacting with water. (a-d) Representative gelatin films cross-linked by 0 mg/mL, 1 mg/mL, 5 mg/mL, and 10 mg/mL cross-linker, respectively.

**
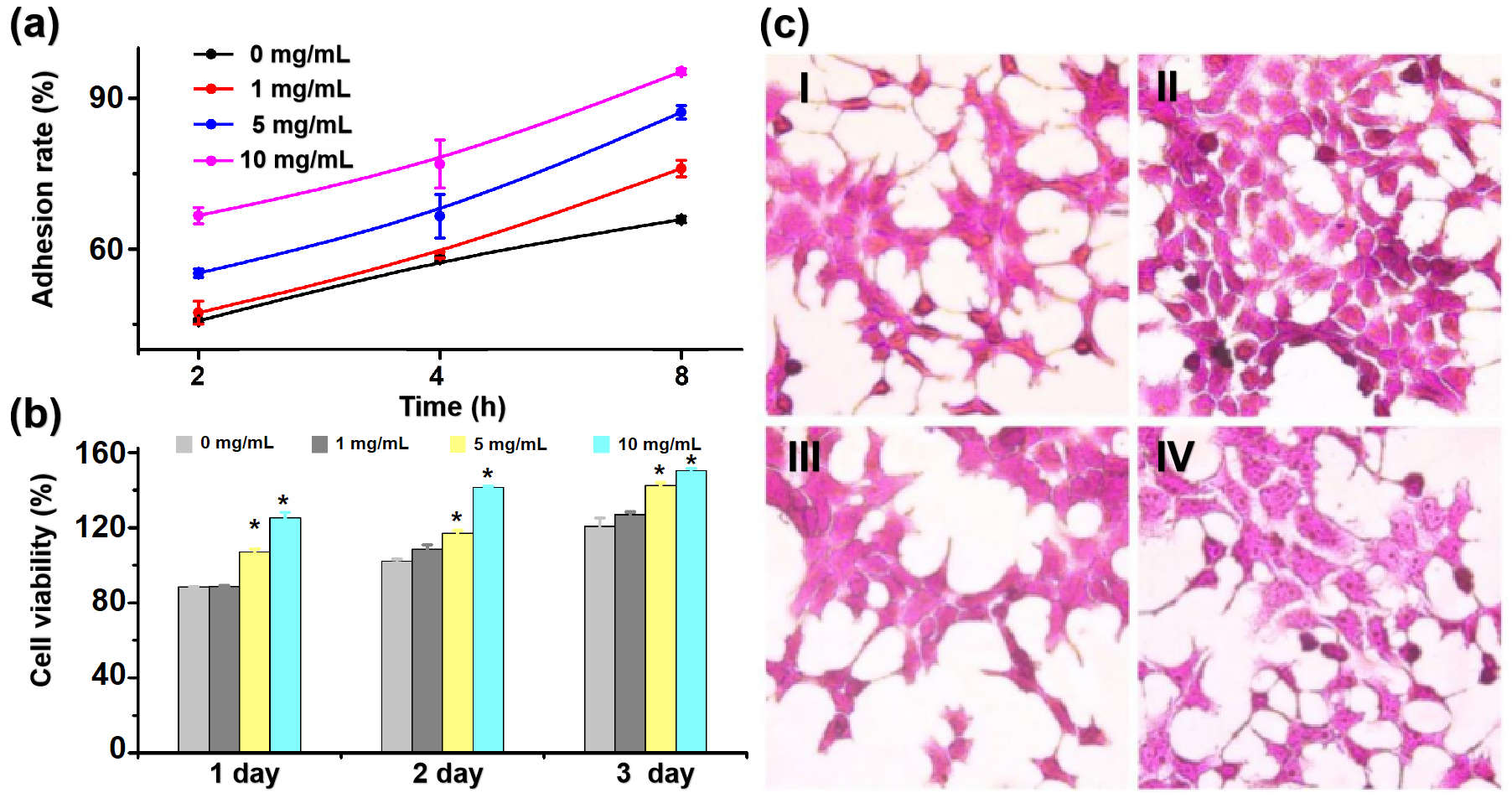
**

**Supplementary Fig. S6** Cell adhesion and cytotoxicity evaluation of the film. (a) Cell adhesion ratio for films cross-linked by NHS-SS-NHS of different concentrations. (b )Proliferation of cells on the gelatin films. For native gelatin (0 mg/mL cross-linker), the cells were grown on TCPS. The differences in cell viability between the native and cross-linked films are significant, as indicated with one asterisk (p<0.05). (c) Morphology of the cells grown on gelatin films cross-linked by 0 mg/mL (I), 1 mg/mL (II), 5 mg/mL (III), and 10 mg/mL (IV) cross-linker.

**Supplementary Fig. S
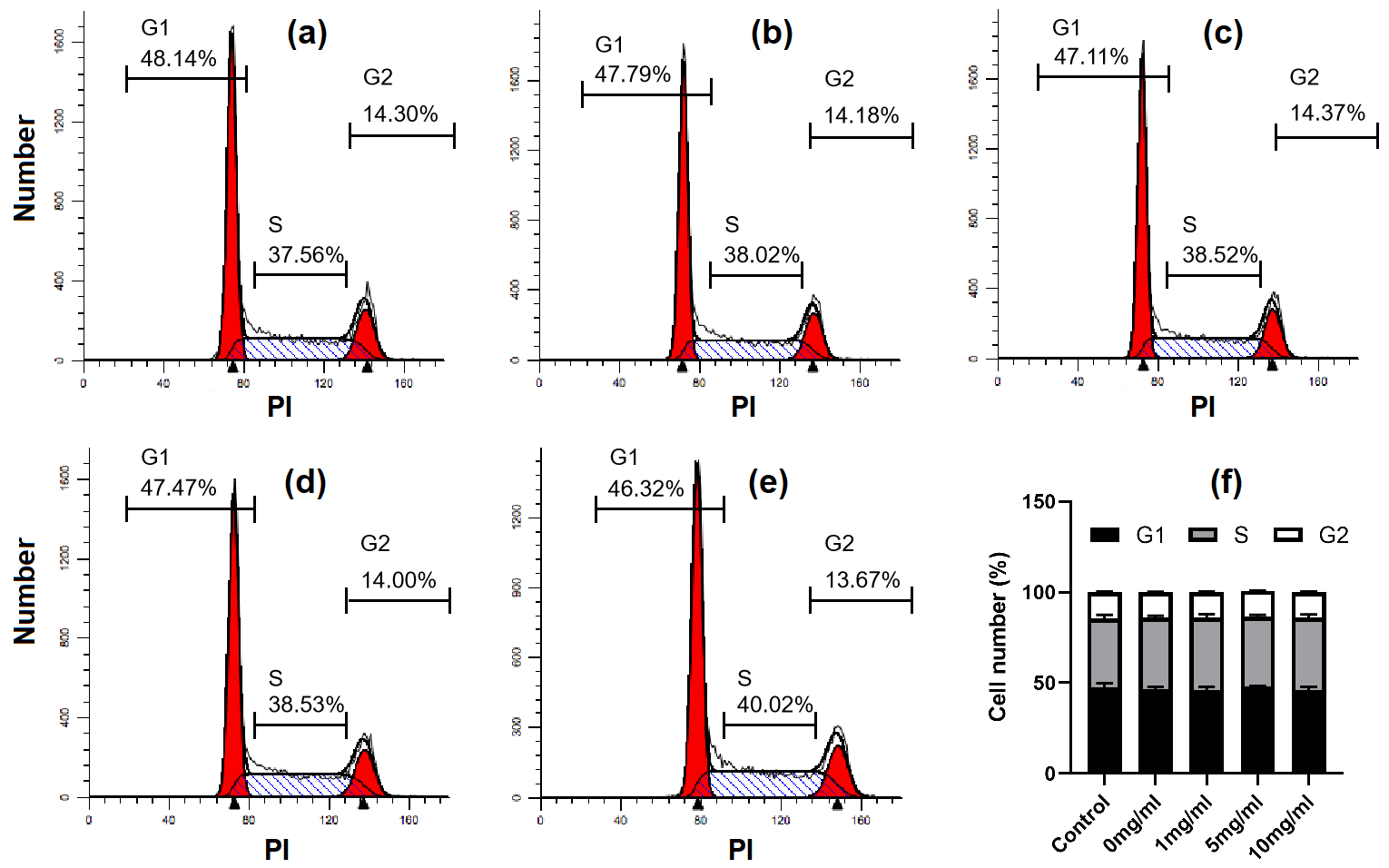
7** Cellular cycle evaluation of the cells grown on the film. (a) Cells were grown on TCPS without gelatin for control. (b) Cells were grown on TCPS with native gelatin. (c-e) Cells were grown on gelatin films cross-linked by 1 mg/mL (c), 5 mg/mL (d), and 10 mg/mL (e) cross-linker, respectively. (f) Comparison in cell numbers for three phases between the films.

**
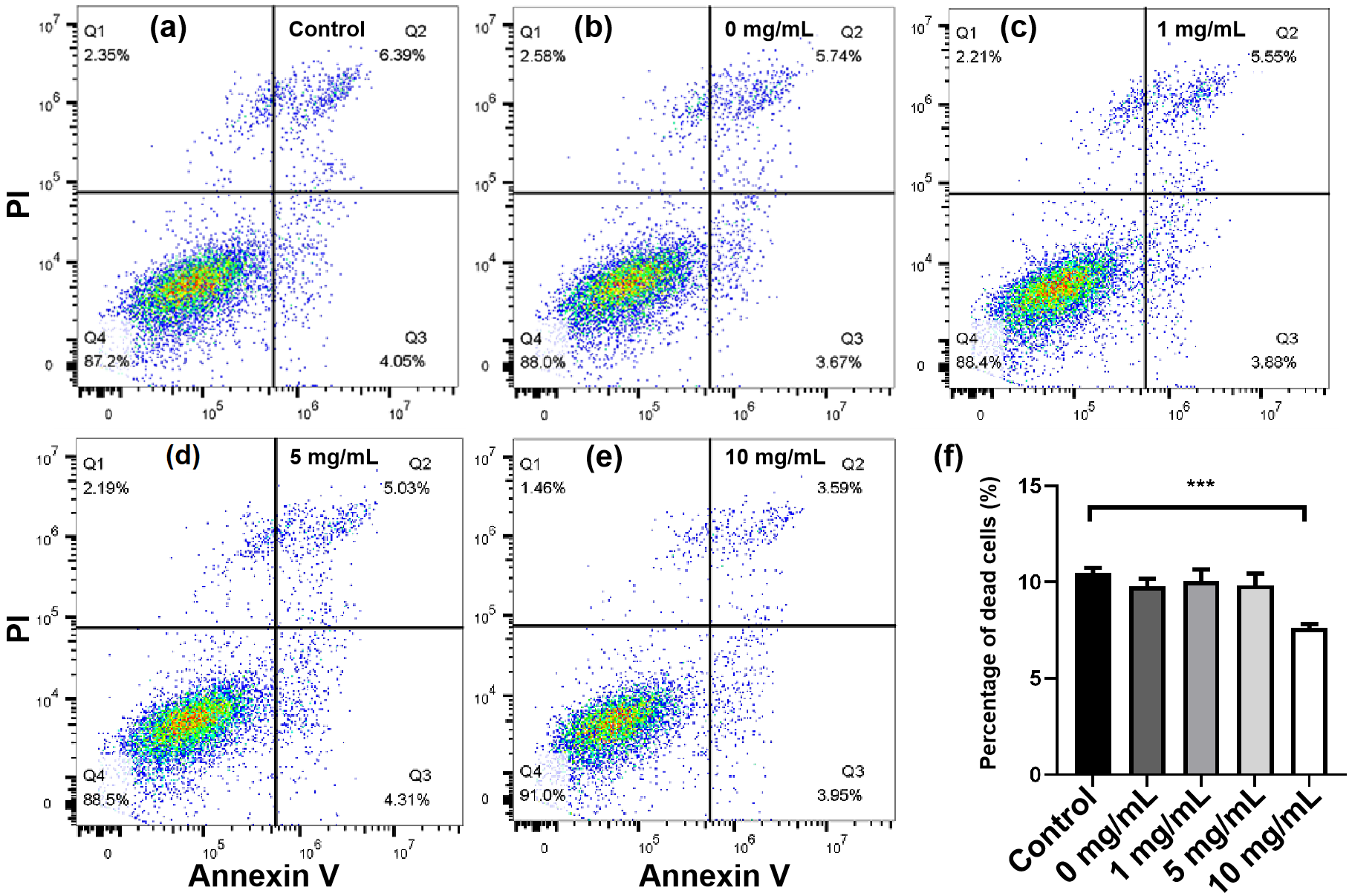
**

**Supplementary Fig. S8** Apoptosis evaluation of the cells grown on the film. (a) Cells were grown on TCPS without gelatin for control. (b) Cells were grown on TCPS with native gelatin. (c-e) Cells were grown on gelatin films cross-linked by 1 mg/mL, 5 mg/mL, and 10 mg/mL cross-linker, respectively. (f) Comparison in dead cell numbers between the films. The differences in dead cell number were indicated with three asterisks (p<0.01).

**Protocol S1.** Bicinchoninic Acid (BCA) Absorbance Assay

Native gelatin dissolved in water was used to create protein standards. The protein solutions of 25 μL were added to 96-well plate with three replications for each sample. BCA working reagent of 200μL was added to each well. The plate was incubated at 37℃ for 30min. Then absorbance at 562 nm was recorded by a microplate reader (Epoch 2, BioTek) at varying intervals throughout the dynamic dissolution of the gelatin films. A standard curve was prepared by plotting each gelatin concentration in μg/mL versus its absorbance at 562 nm.

**Protocol S2. MTT assays**

Pipette 50 μL MTT reagent (Boster, China) to each well of the plate and incubate the plate at 37°C for 4h in the dark, during which purple precipitates (formazan crystals) appeared. Then, 500 μL formanzan solution was added to each well of the plate. The plate was incubated at 37°C until the purple precipitate was completely dissolved. Finally, the plate wells were read at 570 nm on a microplate reader (Bio-Tek, USA). The assays were performed in triplicates. Cell viabilities were calculated by equation: CV=OD_S_/OD_C_, where CV, OD_E_, OD_C_ represent cell viability (%), the absorbance of experimental groups, and the absorbance of control groups, respectively.
